# Supplementary material for: Hot spot prediction in protein-protein interactions by an ensemble system
Source: BMC Syst Biol. 2018 Dec 31;12(Suppl 9):132. doi: 10.1186/s12918-018-0665-8 (PMC6311905; doi:10.1186/s12918-018-0665-8)
Supplement: Supplementary file 2 — All relevant MATLAB code. A simple Matlab implement of our predictor. (ZIP 19,742 kb) [file 12918_2018_665_MOESM2_ESM.zip › Additional file 2/Read ASA/ASA website/dbMPIKT/4BKLA.html]

 NetSurfP - Protein Surface Accessibility and Secondary Structure Predictions

  

|  |  |  |
| --- | --- | --- |
|  |  | NetSurfP - Protein Surface Accessibility and Secondary Structure Predictions  Technical University of Denmark |

  


---

```
# For publication of results, please cite:
# A generic method for assignment of reliability scores applied to solvent accessibility predictions.
# Bent Petersen, Thomas Nordahl Petersen, Pernille Andersen, Morten Nielsen and Claus Lundegaard
# BMC Structural Biology 2009, 9:51 doi:10.1186/1472-6807-9-51
#
# Column 1: Class assignment - B for buried or E for Exposed - Threshold: 25% exposure, but not based on RSA
# Column 2: Amino acid
# Column 3: Sequence name
# Column 4: Amino acid number
# Column 5: Relative Surface Accessibility - RSA
# Column 6: Absolute Surface Accessibility
# Column 7: Z-fit score for RSA prediction
# Column 8: Probability for Alpha-Helix
# Column 9: Probability for Beta-strand
# Column 10: Probability for Coil
E Q  4BKLA                  1    0.813 145.148   1.360   0.003   0.003   0.994
B V  4BKLA                  2    0.194  29.787  -0.283   0.004   0.616   0.381
E Q  4BKLA                  3    0.431  76.923   1.397   0.002   0.816   0.182
B L  4BKLA                  4    0.148  27.044   0.610   0.002   0.816   0.182
E Q  4BKLA                  5    0.402  71.815   0.626   0.021   0.756   0.223
B Q  4BKLA                  6    0.255  45.472   0.715   0.004   0.514   0.481
E S  4BKLA                  7    0.430  50.373  -0.533   0.020   0.205   0.775
E G  4BKLA                  8    0.487  38.358  -1.812   0.018   0.088   0.893
E A  4BKLA                  9    0.513  56.555  -2.140   0.018   0.047   0.935
E E  4BKLA                 10    0.514  89.883  -1.641   0.020   0.205   0.775
E L  4BKLA                 11    0.391  71.610  -0.740   0.022   0.359   0.619
E A  4BKLA                 12    0.303  33.380   0.629   0.021   0.451   0.528
E K  4BKLA                 13    0.419  86.127   1.213   0.020   0.205   0.775
E P  4BKLA                 14    0.450  63.926  -0.457   0.018   0.047   0.935
E G  4BKLA                 15    0.622  48.967  -1.893   0.005   0.045   0.951
E T  4BKLA                 16    0.414  57.450  -0.584   0.004   0.138   0.858
E S  4BKLA                 17    0.423  49.540  -0.261   0.004   0.514   0.481
B V  4BKLA                 18    0.150  23.101   0.893   0.002   0.816   0.182
E K  4BKLA                 19    0.416  85.674   0.981   0.001   0.900   0.099
B M  4BKLA                 20    0.074  14.787   0.467   0.001   0.900   0.099
B S  4BKLA                 21    0.158  18.553   0.855   0.001   0.900   0.099
B C  4BKLA                 22    0.033   4.577  -0.000   0.001   0.900   0.099
B K  4BKLA                 23    0.278  57.143   0.595   0.001   0.900   0.099
B A  4BKLA                 24    0.089   9.841  -0.509   0.002   0.816   0.182
B S  4BKLA                 25    0.165  19.291  -0.412   0.004   0.514   0.481
B G  4BKLA                 26    0.215  16.881  -1.677   0.004   0.197   0.799
B Y  4BKLA                 27    0.226  48.318  -0.698   0.005   0.336   0.660
B T  4BKLA                 28    0.164  22.733  -1.026   0.004   0.514   0.481
B F  4BKLA                 29    0.095  19.046  -0.925   0.021   0.451   0.528
B I  4BKLA                 30    0.222  41.089  -1.158   0.021   0.279   0.699
B S  4BKLA                 31    0.278  32.640  -1.232   0.018   0.088   0.893
B Y  4BKLA                 32    0.099  21.114   0.251   0.019   0.141   0.840
B W  4BKLA                 33    0.088  21.212   0.120   0.021   0.451   0.528
B M  4BKLA                 34    0.029   5.883   0.275   0.021   0.756   0.223
B N  4BKLA                 35    0.047   6.866  -0.266   0.021   0.756   0.223
B W  4BKLA                 36    0.037   8.850   0.304   0.021   0.756   0.223
B V  4BKLA                 37    0.037   5.641   0.310   0.018   0.846   0.136
B K  4BKLA                 38    0.128  26.288   0.357   0.023   0.655   0.322
B Q  4BKLA                 39    0.095  17.003   0.442   0.022   0.359   0.619
B R  4BKLA                 40    0.215  49.327   0.783   0.005   0.045   0.951
E P  4BKLA                 41    0.360  51.098   0.743   0.005   0.015   0.979
E G  4BKLA                 42    0.587  46.221  -1.801   0.018   0.019   0.964
E Q  4BKLA                 43    0.389  69.422   0.574   0.018   0.019   0.964
E G  4BKLA                 44    0.411  32.338  -0.535   0.018   0.088   0.893
B L  4BKLA                 45    0.158  29.003   1.029   0.005   0.336   0.660
B E  4BKLA                 46    0.088  15.443  -0.269   0.004   0.616   0.381
B W  4BKLA                 47    0.043  10.438   0.001   0.001   0.900   0.099
B I  4BKLA                 48    0.025   4.532   0.829   0.001   0.959   0.040
B G  4BKLA                 49    0.018   1.401  -0.121   0.001   0.959   0.040
B A  4BKLA                 50    0.072   7.956  -0.475   0.001   0.959   0.040
B I  4BKLA                 51    0.023   4.163   0.901   0.001   0.959   0.040
B N  4BKLA                 52    0.141  20.686   0.503   0.003   0.718   0.279
B P  4BKLA                 53    0.263  37.348  -1.063   0.005   0.262   0.733
E S  4BKLA                 54    0.534  62.573  -1.779   0.005   0.045   0.951
E D  4BKLA                 55    0.562  81.013  -2.464   0.018   0.019   0.964
E G  4BKLA                 56    0.500  39.334  -2.155   0.018   0.019   0.964
E Y  4BKLA                 57    0.472 100.760  -0.616   0.018   0.047   0.935
E T  4BKLA                 58    0.356  49.433   0.055   0.019   0.141   0.840
E E  4BKLA                 59    0.430  75.104   0.077   0.020   0.205   0.775
B Y  4BKLA                 60    0.187  40.005   0.586   0.064   0.216   0.721
B N  4BKLA                 61    0.202  29.573  -0.190   0.052   0.084   0.864
E Q  4BKLA                 62    0.621 110.821  -0.558   0.053   0.043   0.903
E K  4BKLA                 63    0.426  87.546   0.105   0.018   0.088   0.893
B F  4BKLA                 64    0.083  16.738  -0.162   0.019   0.141   0.840
E K  4BKLA                 65    0.399  82.136   0.777   0.004   0.197   0.799
B D  4BKLA                 66    0.224  32.250  -1.704   0.005   0.336   0.660
B K  4BKLA                 67    0.211  43.485   0.674   0.003   0.718   0.279
B A  4BKLA                 68    0.058   6.359  -0.491   0.001   0.900   0.099
B I  4BKLA                 69    0.135  25.049   0.784   0.001   0.959   0.040
B M  4BKLA                 70    0.049   9.765   1.014   0.001   0.959   0.040
B T  4BKLA                 71    0.113  15.673   0.290   0.002   0.816   0.182
E A  4BKLA                 72    0.266  29.335   0.661   0.004   0.514   0.481
B D  4BKLA                 73    0.295  42.538  -0.646   0.004   0.420   0.576
E R  4BKLA                 74    0.442 101.241  -0.120   0.018   0.088   0.893
B S  4BKLA                 75    0.264  30.953  -1.818   0.018   0.047   0.935
E S  4BKLA                 76    0.509  59.713   0.777   0.019   0.141   0.840
B S  4BKLA                 77    0.251  29.429  -0.406   0.022   0.359   0.619
B T  4BKLA                 78    0.189  26.214   0.889   0.018   0.846   0.136
B A  4BKLA                 79    0.068   7.505  -0.796   0.011   0.918   0.071
B Y  4BKLA                 80    0.159  34.085   0.478   0.006   0.962   0.032
B M  4BKLA                 81    0.044   8.724   0.694   0.001   0.959   0.040
B Q  4BKLA                 82    0.149  26.522  -0.282   0.001   0.900   0.099
B L  4BKLA                 83    0.078  14.209  -0.800   0.003   0.718   0.279
E S  4BKLA                 84    0.354  41.524  -0.052   0.004   0.420   0.576
E S  4BKLA                 85    0.401  47.032  -0.965   0.004   0.197   0.799
B L  4BKLA                 86    0.127  23.235   0.001   0.004   0.085   0.910
E T  4BKLA                 87    0.466  64.606   0.995   0.018   0.047   0.935
E S  4BKLA                 88    0.360  42.251  -0.765   0.052   0.084   0.864
E E  4BKLA                 89    0.461  80.484  -0.574   0.053   0.043   0.903
B D  4BKLA                 90    0.170  24.540  -0.532   0.018   0.088   0.893
B S  4BKLA                 91    0.095  11.157   0.522   0.021   0.451   0.528
B A  4BKLA                 92    0.033   3.681  -0.300   0.018   0.846   0.136
B L  4BKLA                 93    0.059  10.803   1.105   0.011   0.918   0.071
B Y  4BKLA                 94    0.031   6.539   1.026   0.011   0.918   0.071
B Y  4BKLA                 95    0.051  10.941   0.698   0.018   0.846   0.136
B C  4BKLA                 96    0.016   2.246   1.440   0.021   0.756   0.223
B A  4BKLA                 97    0.030   3.295   0.175   0.022   0.552   0.426
B R  4BKLA                 98    0.165  37.762   0.398   0.021   0.279   0.699
E Y  4BKLA                 99    0.389  83.022  -0.288   0.018   0.088   0.893
E G  4BKLA                100    0.331  26.034  -0.959   0.018   0.047   0.935
B G  4BKLA                101    0.296  23.295  -1.608   0.018   0.047   0.935
B Y  4BKLA                102    0.108  22.994  -0.963   0.020   0.205   0.775
B F  4BKLA                103    0.064  12.925  -0.076   0.022   0.359   0.619
B D  4BKLA                104    0.060   8.603  -0.074   0.022   0.359   0.619
B Y  4BKLA                105    0.053  11.412  -0.263   0.022   0.359   0.619
B W  4BKLA                106    0.042  10.077  -0.354   0.021   0.279   0.699
B G  4BKLA                107    0.043   3.416  -2.992   0.021   0.279   0.699
E Q  4BKLA                108    0.312  55.670  -0.759   0.019   0.141   0.840
E G  4BKLA                109    0.270  21.280  -1.276   0.018   0.047   0.935
E T  4BKLA                110    0.341  47.241  -0.798   0.018   0.047   0.935
E T  4BKLA                111    0.390  54.051  -0.777   0.019   0.141   0.840
B L  4BKLA                112    0.162  29.571  -1.642   0.018   0.088   0.893
E T  4BKLA                113    0.342  47.394  -1.457   0.019   0.141   0.840
B V  4BKLA                114    0.177  27.128  -1.333   0.020   0.205   0.775
B S  4BKLA                115    0.253  29.640  -1.697   0.018   0.088   0.893
E S  4BKLA                116    0.398  46.657  -2.326   0.005   0.045   0.951
E A  4BKLA                117    0.331  36.443  -2.215   0.005   0.015   0.979
E K  4BKLA                118    0.514 105.668  -1.131   0.005   0.015   0.979
E T  4BKLA                119    0.477  66.118  -2.040   0.005   0.015   0.979
E T  4BKLA                120    0.426  59.031  -2.071   0.005   0.015   0.979
E P  4BKLA                121    0.298  42.272  -2.240   0.005   0.015   0.979
B P  4BKLA                122    0.242  34.354  -2.296   0.005   0.015   0.979
E S  4BKLA                123    0.415  48.661  -1.790   0.019   0.141   0.840
E V  4BKLA                124    0.465  71.409  -1.859   0.020   0.205   0.775
E Y  4BKLA                125    0.366  78.300  -1.838   0.019   0.141   0.840
E P  4BKLA                126    0.523  74.214  -2.222   0.020   0.205   0.775
E L  4BKLA                127    0.459  84.043  -1.677   0.020   0.205   0.775
E A  4BKLA                128    0.346  38.096  -1.315   0.004   0.085   0.910
E P  4BKLA                129    0.402  57.058  -1.978   0.005   0.045   0.951
E G  4BKLA                130    0.364  28.663  -2.540   0.005   0.045   0.951
E C  4BKLA                131    0.343  48.227  -2.084   0.005   0.015   0.979
E G  4BKLA                132    0.569  44.749  -2.297   0.005   0.015   0.979
E D  4BKLA                133    0.484  69.687  -2.536   0.005   0.015   0.979
E T  4BKLA                134    0.494  68.559  -1.921   0.018   0.019   0.964
E T  4BKLA                135    0.607  84.135  -1.924   0.018   0.019   0.964
E G  4BKLA                136    0.653  51.360  -2.372   0.005   0.015   0.979
E S  4BKLA                137    0.487  57.030  -1.796   0.005   0.045   0.951
E S  4BKLA                138    0.364  42.649   0.223   0.005   0.336   0.660
B V  4BKLA                139    0.077  11.758   0.628   0.002   0.816   0.182
B T  4BKLA                140    0.134  18.530   0.071   0.001   0.900   0.099
B L  4BKLA                141    0.040   7.342   0.040   0.001   0.959   0.040
B G  4BKLA                142    0.043   3.416  -0.384   0.001   0.959   0.040
B C  4BKLA                143    0.023   3.285   0.872   0.001   0.900   0.099
B L  4BKLA                144    0.140  25.616   0.691   0.001   0.900   0.099
B V  4BKLA                145    0.038   5.871  -0.746   0.002   0.816   0.182
E K  4BKLA                146    0.264  54.284   0.245   0.004   0.514   0.481
E G  4BKLA                147    0.390  30.677  -2.003   0.004   0.197   0.799
B Y  4BKLA                148    0.237  50.732  -1.194   0.004   0.197   0.799
E F  4BKLA                149    0.300  60.150  -0.569   0.005   0.262   0.733
B P  4BKLA                150    0.251  35.631  -1.385   0.004   0.197   0.799
E E  4BKLA                151    0.557  97.273  -1.054   0.004   0.138   0.858
E S  4BKLA                152    0.391  45.778  -1.421   0.005   0.262   0.733
B V  4BKLA                153    0.152  23.409  -0.356   0.003   0.718   0.279
B T  4BKLA                154    0.238  32.983  -0.539   0.001   0.900   0.099
B V  4BKLA                155    0.057   8.776   0.240   0.001   0.959   0.040
B T  4BKLA                156    0.192  26.575   0.030   0.001   0.900   0.099
B W  4BKLA                157    0.055  13.179  -0.406   0.011   0.918   0.071
E S  4BKLA                158    0.270  31.691   0.087   0.002   0.816   0.182
E S  4BKLA                159    0.381  44.712  -0.557   0.004   0.514   0.481
E G  4BKLA                160    0.463  36.399  -0.423   0.018   0.088   0.893
E S  4BKLA                161    0.535  62.655  -0.075   0.018   0.019   0.964
E L  4BKLA                162    0.507  92.905  -1.294   0.018   0.047   0.935
E S  4BKLA                163    0.581  68.105  -2.329   0.018   0.088   0.893
E S  4BKLA                164    0.560  65.609  -0.553   0.018   0.088   0.893
E S  4BKLA                165    0.474  55.565  -1.162   0.019   0.141   0.840
B V  4BKLA                166    0.239  36.765  -1.367   0.022   0.359   0.619
E H  4BKLA                167    0.381  69.358  -0.167   0.003   0.718   0.279
B T  4BKLA                168    0.296  41.069  -0.841   0.021   0.756   0.223
B F  4BKLA                169    0.246  49.272   0.891   0.022   0.552   0.426
B P  4BKLA                170    0.230  32.594  -0.134   0.022   0.359   0.619
B A  4BKLA                171    0.187  20.552  -2.624   0.021   0.451   0.528
B L  4BKLA                172    0.271  49.712  -1.194   0.022   0.552   0.426
E L  4BKLA                173    0.373  68.278  -1.294   0.021   0.451   0.528
E Q  4BKLA                174    0.496  88.657  -0.797   0.005   0.262   0.733
E S  4BKLA                175    0.731  85.626  -1.509   0.005   0.015   0.979
E G  4BKLA                176    0.503  39.594  -2.327   0.005   0.045   0.951
E L  4BKLA                177    0.409  74.888   0.394   0.004   0.420   0.576
B Y  4BKLA                178    0.147  31.371  -0.710   0.003   0.718   0.279
B T  4BKLA                179    0.188  26.034  -0.989   0.002   0.816   0.182
B M  4BKLA                180    0.146  29.135  -1.190   0.001   0.900   0.099
B S  4BKLA                181    0.157  18.377  -0.748   0.001   0.900   0.099
B S  4BKLA                182    0.059   6.891  -1.094   0.001   0.900   0.099
B S  4BKLA                183    0.254  29.781   0.065   0.001   0.900   0.099
B V  4BKLA                184    0.060   9.283   0.152   0.001   0.900   0.099
E T  4BKLA                185    0.310  43.011   0.592   0.001   0.900   0.099
B V  4BKLA                186    0.065   9.929   0.316   0.002   0.816   0.182
E P  4BKLA                187    0.382  54.177   1.012   0.021   0.451   0.528
E S  4BKLA                188    0.404  47.337  -0.574   0.020   0.205   0.775
E S  4BKLA                189    0.602  70.543  -1.041   0.005   0.336   0.660
E T  4BKLA                190    0.607  84.177   0.653   0.005   0.262   0.733
B W  4BKLA                191    0.162  39.033  -0.712   0.004   0.138   0.858
E P  4BKLA                192    0.745 105.687   0.947   0.005   0.045   0.951
E S  4BKLA                193    0.762  89.248   1.020   0.005   0.015   0.979
E Q  4BKLA                194    0.544  97.212   0.760   0.005   0.262   0.733
E T  4BKLA                195    0.510  70.737   1.294   0.002   0.816   0.182
B V  4BKLA                196    0.069  10.575   0.897   0.001   0.959   0.040
E T  4BKLA                197    0.314  43.580   2.008   0.001   0.959   0.040
B C  4BKLA                198    0.028   3.973   0.200   0.001   0.959   0.040
E S  4BKLA                199    0.355  41.547   0.923   0.001   0.959   0.040
B V  4BKLA                200    0.107  16.384  -0.172   0.001   0.900   0.099
E A  4BKLA                201    0.515  56.764   0.391   0.001   0.900   0.099
B H  4BKLA                202    0.197  35.852   0.194   0.004   0.616   0.381
E P  4BKLA                203    0.643  91.213   0.064   0.005   0.045   0.951
E A  4BKLA                204    0.750  82.694  -1.875   0.005   0.015   0.979
E S  4BKLA                205    0.605  70.929  -0.994   0.005   0.045   0.951
E S  4BKLA                206    0.571  66.933  -0.356   0.004   0.085   0.910
E T  4BKLA                207    0.400  55.522   0.561   0.004   0.420   0.576
E T  4BKLA                208    0.502  69.600   1.220   0.002   0.816   0.182
B V  4BKLA                209    0.112  17.276   0.168   0.001   0.900   0.099
E D  4BKLA                210    0.656  94.472   0.381   0.001   0.900   0.099
B K  4BKLA                211    0.208  42.847   1.046   0.002   0.816   0.182
E K  4BKLA                212    0.557 114.534   0.638   0.002   0.816   0.182
B L  4BKLA                213    0.169  31.017   0.327   0.004   0.616   0.381
E E  4BKLA                214    0.586 102.304   0.389   0.005   0.336   0.660
E P  4BKLA                215    0.402  57.101  -0.936   0.018   0.088   0.893
E S  4BKLA                216    0.689  80.739  -0.491   0.004   0.085   0.910
E G  4BKLA                217    0.432  34.006  -1.379   0.005   0.045   0.951
E P  4BKLA                218    0.301  42.740  -0.505   0.005   0.015   0.979
E I  4BKLA                219    0.596 110.334  -0.879   0.005   0.045   0.951
E S  4BKLA                220    0.332  38.934  -1.104   0.004   0.197   0.799
E T  4BKLA                221    0.466  64.620  -0.657   0.005   0.336   0.660
E I  4BKLA                222    0.322  59.588  -0.890   0.005   0.336   0.660
E N  4BKLA                223    0.398  58.282  -1.096   0.005   0.262   0.733
E P  4BKLA                224    0.322  45.763  -1.240   0.004   0.085   0.910
E C  4BKLA                225    0.352  49.477  -0.865   0.005   0.015   0.979
B P  4BKLA                226    0.286  40.612  -1.143   0.003   0.003   0.994
E P  4BKLA                227    0.422  59.839  -1.166   0.003   0.003   0.994
B C  4BKLA                228    0.223  31.267  -0.866   0.005   0.015   0.979
E K  4BKLA                229    0.674 138.601   0.139   0.018   0.019   0.964
E E  4BKLA                230    0.697 121.801  -0.920   0.018   0.019   0.964
E C  4BKLA                231    0.641  89.996  -2.342   0.003   0.003   0.994

Explain the output.  Go back.
```

---
